# Methanolic Extracts of Bitter Melon Inhibit Colon Cancer Stem Cells by Affecting Energy Homeostasis and Autophagy

**DOI:** 10.1155/2013/702869

**Published:** 2013-03-06

**Authors:** Deep Kwatra, Dharmalingam Subramaniam, Prabhu Ramamoorthy, David Standing, Elizabeth Moran, Ravichandiran Velayutham, Ashim Mitra, Shahid Umar, Shrikant Anant

**Affiliations:** ^1^Department of Molecular and Integrative Physiology, University of Kansas Medical Center, 3901 Rainbow Boulevard MS 3040, Kansas City, KS 66160, USA; ^2^University of Kansas Cancer Center, University of Kansas Medical Center, 3901 Rainbow Boulevard MS 3040, Kansas City, KS 66160, USA; ^3^Department of Pharmacognosy, Vels University, Pallavaram, Chennai 600117, India; ^4^Department of Pharmaceutical Sciences, University of Missouri at Kansas City, Kansas City, MO 64108, USA

## Abstract

Bitter melon fruit is recommended in ancient Indian and Chinese medicine for prevention/treatment of diabetes. However its effects on cancer progression are not well understood. Here, we have determined the efficacy of methanolic extracts of bitter melon on colon cancer stem and progenitor cells. Both, whole fruit (BMW) and skin (BMSk) extracts showed significant inhibition of cell proliferation and colony formation, with BMW showing greater efficacy. In addition, the cells were arrested at the S phase of cell cycle. Moreover, BMW induced the cleavage of LC3B but not caspase 3/7, suggesting that the cells were undergoing autophagy and not apoptosis. Further confirmation of autophagy was obtained when western blots showed reduced Bcl-2 and increased Beclin-1, Atg 7 and 12 upon BMW treatment. BMW reduced cellular ATP levels coupled with activation of AMP activated protein kinase; on the other hand, exogenous additions of ATP lead to revival of cell proliferation. Finally, BMW treatment results in a dose-dependent reduction in the number and size of colonospheres. The extracts also decreased the expression of DCLK1 and Lgr5, markers of quiescent, and activated stem cells. Taken together, these results suggest that the extracts of bitter melon can be an effective preventive/therapeutic agent for colon cancer.

## 1. Introduction

Colorectal cancer is the second leading cause of cancer related deaths and the third most commonly occurring noncutaneous carcinoma in the United States of America [1]. Although early diagnosis often leads to a complete cure, in most cases the polyps go undetected. In such cases, therapies such as surgical intervention, chemotherapy, and radiation are often not sufficient to tackle the disease, thus needing other prevention-related or nonconventional therapeutic strategies. Hence there is a need of better options for therapy and prevention of the disease. 

A number of studies have shown that diet can play a significant role in the development of colon cancer as a higher risk is associated with consumption of high-fat, low-fiber diet and red meat [2, 3]. Use of certain foods and condiments (such as curcumin) in food may be responsible for lower prevalence of the disease in certain parts of India and central Asia [4, 5]. Bitter melon (*Momordica charantia*) is a tropical and subtropical vine, widely grown in Asia, Africa, and the Caribbean for its edible fruit. The fruit is recommended in ancient Indian and Chinese medicine for prevention/treatment of diabetes [6, 7], though all parts of the plant (fruit, seed, and leaves) have been shown to possess hypoglycemic properties [8]. Studies have shown that bitter melon extracts are well tolerated in both acute and chronic doses in animals [9–11]. Recent studies have demonstrated that aqueous extracts of bitter melon can inhibit the growth of breast and prostate cancers [12–14]. Though the mechanisms behind the antitumor activity are poorly known, the previous studies hint towards induction of apoptosis as one potential mechanism [15, 16]. 

Not much is known about the active principles in bitter melon that have anticancer activity. A 30 kDa ribosome-inactivating protein (RIP) termed Momordica or MAP30 was identified in bitter melon. Addition of MAP30 to *MDA-MB-231* breast cancer cells in culture reduced proliferation of the cells. In addition, there was a reduction in expression of Human Epidermal Growth Factor Receptor 2 (HER2) [15–17]. Moreover, modification of MAP30 resulted in reduced immunogenicity while retaining the anti-proliferative activity against prostate cancer cell xenografts in nude mice [15, 16]. Thirteen cucurbitane-type triterpene glycosides have also been identified in bitter melon extracts with potential anti-proliferative activity [18]. *α*-eleostearic acid is found in high amounts in the seeds of bitter melon and may be effective in some (MDA-MB-231, ER +ve MDA-ER*α*7) tumor cells but not in others [19, 20]. However, none of these studies have been comprehensive, and no major studies have been performed using colon cancer cells.

In this paper, we present the results of our *in vitro* experiments showing that methanolic extracts of bitter melon (BMW) inhibit cell proliferation, prevent colony formation, and promote S phase cell cycle arrest of colon cancer cells. We also show that these extracts suppress cancer cell spheroid formation suggesting that the extracts target stem cells within the cancer. Mechanistically, we have determined that while the extracts do not induce apoptosis, there is autophagy via the AMPK signaling pathway. In addition, the extracts modulate energy homeostasis to affect the viability of the colon cancer cells.

## 2. Materials and Methods

### 2.1. Cell Culture and Preparation of Bitter Melon Extracts (BMW)

HT-29, SW480, and human foreskin fibroblast (HFF) cells (all from American Type Culture Collection, Manassas, VA, USA) were grown in Dulbecco's modified eagle medium containing 10% heat inactivated fetal bovine serum (Sigma Aldrich, St. Louis, MO, USA) and 2% antibiotic-antimycotic solution (Mediatech Inc., Herndon, VA, USA) at 37°C in a humidified atmosphere with 5% CO_2_. Methanol extracts of bitter melon skin and whole fruit were prepared from the raw and green variety of young bitter melons (*Momordica charantia* Linn, subcontinent variety). Firstly, for the bitter melon whole fruit extracts (BMW), preweighed amount of fruits was finely chopped and placed in 1 : 1 w/v methanol for 72 h at 4°C. These were then homogenized, centrifuged and the supernatant freeze dried at −45°C for 72 h and stored at −80°C. Similar process was performed to form the skin extracts (BMSk) where the skin was peeled using a peeler till the white flesh was visible, chopped, and then soaked in 1 : 1 w/v of methanol. These dried extracts were dissolved in DMSO to prepare 100 mg/mL stocks, which were utilized for further experiments. To limit batch-to-batch variation, the weight of the final extract was measured and the batches with more than 10% variation in extraction efficiency versus the initial weight of the fruit were discarded. Among the selected batches of the methanolic extract no significant batch-to-batch variations were observed based on proliferation assays.

### 2.2. Proliferation and Apoptosis Assays

To assess the effect of BMW on proliferation, 5,000 cells per well were seeded on to 96-well plates and grown overnight. The cells were then treated with increasing doses of BMW and BMSk in DMEM media containing 10% FBS. Analysis of cell proliferation after the treatment period was estimated by the hexosaminidase assay as previously described [21]. Briefly, the medium was removed and hexosaminidase substrate solution in citrate buffer pH 5 (7.5 mM), p-nitrophenol-N-acetyl-beta-D-glucosaminidase (Sigma Aldrich) was added at 75 *μ*L per well. The plate was incubated at 37°C in 100% humidity for 30 minutes. The reaction was stopped with 112.5 *μ*L of 50 mM glycine containing 5 mM of EDTA (pH 10.4). The absorbance was measured at 405 nm. Experiments were conducted at *n* = 6, and repeated at least three times. The data were analyzed as percent of control, where the control wells were treated with equivalent amounts of DMSO alone, and the analyzed data was presented as average ± standard error of mean. The differences among mean values were deemed significant at *P* < 0.05. For IC_50_ calculations, a plot between the drug concentration and hexosaminidase activity was generated and the data was fitted either linearly or exponentially. The best fit was used for further processing of data. IC_50_ was obtained by determining the concentration of compounds resulting in 50% of cell death after 48 h of treatment by using GraphPad PRISM software (GraphPad Software, Inc.). For apoptosis, caspase 3/7 activity was measured using the Apo-one Homogeneous Caspase-3/7 Assay kit (Promega, Madison, WI, USA) using manufacturers protocol.

### 2.3. Clonogenicity Assay

6-well plates were seeded with 500 viable cells and were allowed to grow for overnight. The cells were then incubated in the presence or absence of various concentrations (0.5x, 1x, and 2x of IC_50_ calculated from cell proliferation assay) of BMW and BMSk for 48 h. The BMW containing medium was then removed, and the cells were washed in PBS and incubated for an additional 10 d in complete medium. Each treatment was done in triplicate. The colonies obtained were washed with PBS and fixed in 10% formalin for 10 min at room temperature and then washed with PBS followed by staining with hematoxylin. The colonies were compared to untreated cells.

### 2.4. Cell Cycle Analyses

Cells were treated with bitter melon extracts for 24- and 48 h, and subsequently trypsinized and suspended in phosphate buffered saline (PBS). Single-cell suspensions were fixed using 70% ethanol for 2 h, and subsequently permeabilized with PBS containing 1 mg/mL propidium iodide (Sigma-Aldrich), 0.1% Triton X-100 (Sigma-Aldrich), and 2 mg DNase-free RNase (Sigma-Aldrich) at room temperature. Flow cytometry was done with a FACSCalibur analyzer (Becton Dickinson, Mountain, View, CA, USA), capturing 50,000 events for each sample. Results were analyzed with ModFit LT software (Verity Software House, Topsham, ME).

### 2.5. Western Blot Analysis

Cell lysates were subjected to polyacrylamide gel electrophoresis and blotted onto Immobilon polyvinylidene difluoride membranes (Millipore, Bedford, MA, USA). Antibodies were purchased from Cell Signaling Technology (Beverly, MA, USA), Abcam Inc. (Cambridge, MA, USA) and Santa Cruz Biotechnology Inc. (Santa Cruz, CA, USA) and specific proteins were detected by the enhanced chemiluminescence system (GE Healthcare, Piscataway, NJ, USA).

### 2.6. Immunocytochemistry

Cells were plated onto coverslip and allowed to grow for 24 h. The cells were then treated with various concentrations of bitter melon extracts for 48 h. The cells were then fixed with 10% buffered formalin for 10 min and subsequently washed with PBS. The cells were then permeabilized with PBS containing 0.5% Triton X-100 for 10 min. The coverslips were incubated with rabbit anti-LC3B antibody (Abcam Inc., Cambridge, MA, USA), followed by Cyanine Dye (Cy3) labeled anti-rabbit IgG. The slides were further processed using Vectashield ABC kit (Vector Laboratories, Burlingame, CA, USA) followed by DAB staining.

### 2.7. Transmission Electron Microscopy

TEM was performed after 24 h treatment with 100 *μ*g/mL of bitter melon extracts. For positive control, cells were treated with 25 *μ*M rapamycin for 12 h. The cells were fixed in 0.1 M sodium cacodylate buffer containing 4% paraformaldehyde (PFA) and 2% glutaraldehyde for 4 h at room temperature. The samples were postfixed in 1% osmium tetroxide for 1.5 h and washed with 0.1 M sodium cacodylate buffer, followed by dehydration in an ethanol series of 50%, 60%, 75%, 85%, and 95% for 15 minutes each. The cells were washed twice in 100% Ethanol and passed through a series of epon-araldite (6.2 g epon + 4.4 g araldite + 12.4 g of dodecenyl succinic anhydride + 0.8 g N,N-dimethylbenzylamine) solution in ethanol, before embedding the cells in epon-araldite resin. Finally, 100 nm sections were cut using a microtome, and the sections were placed on glow-discharged 300 mesh copper grids. The ultrasections were stained with Sato's lead (mixture of calcinated lead citrate, lead nitrate, lead acetate, and sodium citrate), and observed by a Hitachi H-7600 Transmission Electron Microscope at 2500x.

### 2.8. Monodansylcadaverine Incorporation

Exponentially growing cells were plated onto 96-well plates, cultured for 24 h and cells were treated with increasing concentrations of bitter melon extracts (0–150 *μ*g/mL) for 24 h. Autophagic vacuoles were labeled with monodansylcadaverine (MDC) (Sigma Aldrich) by incubating cells with 0.001 mmol/L MDC in DMEM at 37°C for 10 min. After incubation, cells were washed three times with PBS and immediately analyzed in plate reader for fluorescence at excitation wavelength 380 nm and emission wavelength of 450 nm. Fluorescence measurements of treatment samples were normalized to control.

### 2.9. ATP Determination

Cells were grown on 96-well tissue culture plates and supplemented with culture medium containing different concentrations of bitter melon extracts or AICAR (an AMPK activator). These plates were grown for 1 to 4 d. Lysis solution was added to each set and kept for 10 h. The lysate from these sets was used for quantitative determination of ATP using ATP Determination Kit (Invitrogen). Luminescence was measured using a 96-well microtiter plate reader. Protein content of each well was also measured using Bradford Protein dye and quantified using the same 96-well microtiter plate reader. ATP measurement was normalized to protein content in each well and represented as percentage of control.

### 2.10. Spheroid Assay

For formation of spheroids, cells were cultured in RPMI 1640 (Mediatech) supplemented with 20 ng/mL bFGF (Invitrogen) 10 mL per 500 mL of 50X B27 supplement (Invitrogen) EGF 20 ng/mL (Invitrogen) and antibiotic and antimycotic solution. Cells were seeded at low densities (5000 cells/mL) in 6-well low adhesion plates. The cells were treated with increasing concentrations of BMW (0–150 *μ*g/mL). After 7 days the spheroids were photographed.

### 2.11. DCLK Flow Cytometry

The cells upon treatment with different concentrations of bitter melon extracts and trypsinization were incubated with 1 : 100 dilution of Phycoerythrin (Invitrogen) conjugated DCLK antibody (Abcam) for 30 minutes. The cells were washed twice with PBS containing 10% serum and flow cytometry was done with a FACSCalibur analyzer (Becton Dickinson, Mountain, View, CA, USA), capturing 10,000 events for each sample.

### 2.12. Statistical Analysis

All experiments were conducted at least in triplicate and results were expressed as mean ± SD. Statistical comparisons of mean values were evaluated by Student's *t*-test using GraphPad InStat version 3.1 (GraphPad, La Jolla, CA). Values of *P* < 0.05 were considered significant.

## 3. Results

### 3.1. Bitter Melon Extracts Inhibit the Proliferation and Colony Formation of Colon Cancer Cells

**Figure 1:**
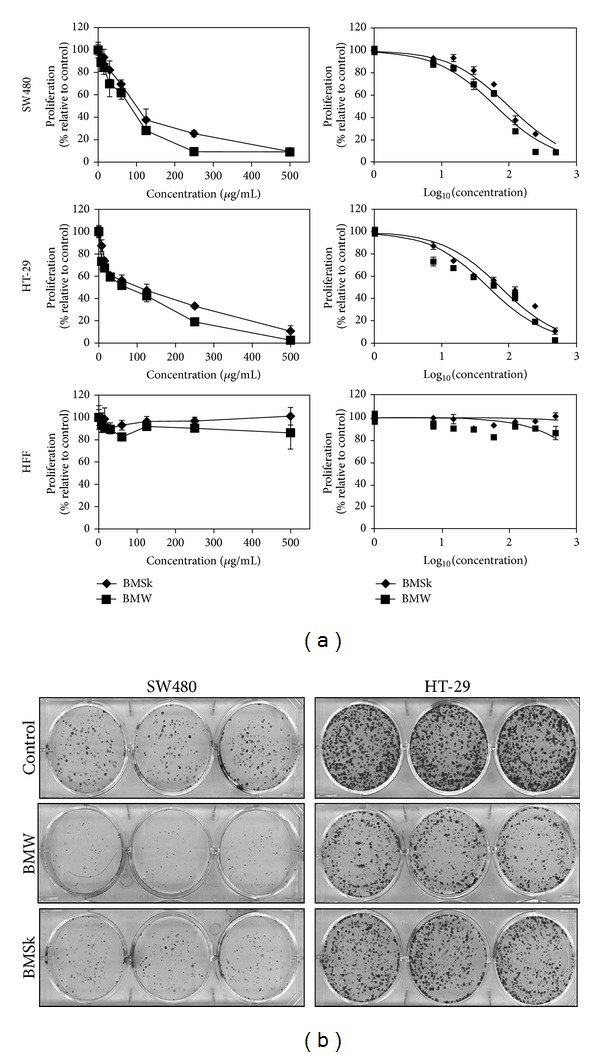
Bitter melon extract inhibits colon cancer cell proliferation and clonogenicity. (a) Bitter melon extract inhibits proliferation of colon cancer cells. Cells were incubated with increasing doses (0–500 *μ*g/mL) of bitter melon whole fruit (BMW) or skin (BMSk) extracts for 48 h and analyzed for cell proliferation. Bitter melon extract treatment resulted in a significant dose-dependent decrease in cell proliferation in both HT-29 and SW480 cells when compared with untreated controls. BMW is better in inhibiting cell proliferation. The extracts did not affect the proliferation of human foreskin fibroblast (HFF) cells suggesting a lack of toxicity in normal cells. (b) Bitter melon extract affects clonogenicity. Colon cancer cells were incubated with 100 *μ*g/mL of bitter melon extract for 48 h and subsequently allowed to grow into colonies for 7 days. Incubation with bitter melon extract inhibits colony formation. Results are representative of three independent experiments.

We first determined the sensitivity of HT-29 and SW480 colon cancer cells to bitter melon extracts. To determine whether bitter melon extracts affect cell proliferation, the cells were grown in the presence of varying concentrations of BMW and BMSk (0–500 *μ*g/mL) for 48 h and the cell proliferation was measured by hexoseminidase assay. Both BMW and BMSk inhibited the proliferation of HT-29 and SW480 cells in a dose-dependent manner (Figure 1(a)). The IC_50_ dose of BMW for proliferation of HT-29 cells was observed to be 57 *μ*g/mL, which was much less than that observed for BMSk (105 *μ*g/mL). Similar trend was observed in SW480 cells where IC_50_ dose of BMW for proliferation was 85 *μ*g/mL, which was again found to be much lower than IC_50_ for BMSk (108 *μ*g/ML) (Figure 1(a)). Under similar conditions, the IC_50_ for the noncancerous human foreskin fibroblast (HFF) cells could not be calculated as neither BMW nor BMSk significantly inhibited proliferation of the cells when compared to controls (Figure 1(b)).

To further study the effect of BMW on cell growth and proliferation in colon cancer cells, clonogenicity assay was performed. SW480 and HT-29 cells were treated with 100 *μ*g/mL BMW and BMSK extracts. Both extracts reduced colony formation of the two cell lines in a concentration dependent manner (Figure 1(b)). However, there was a significantly less number of surviving colonies following BMW treatment as compared to BMSk treated samples, suggesting that BMW was more potent in inhibiting the growth of colon cancer cells (Figure 1(b)).

### 3.2. Bitter Melon Extracts Cause S and G2/M Cell Cycle Arrest

**Figure 2:**
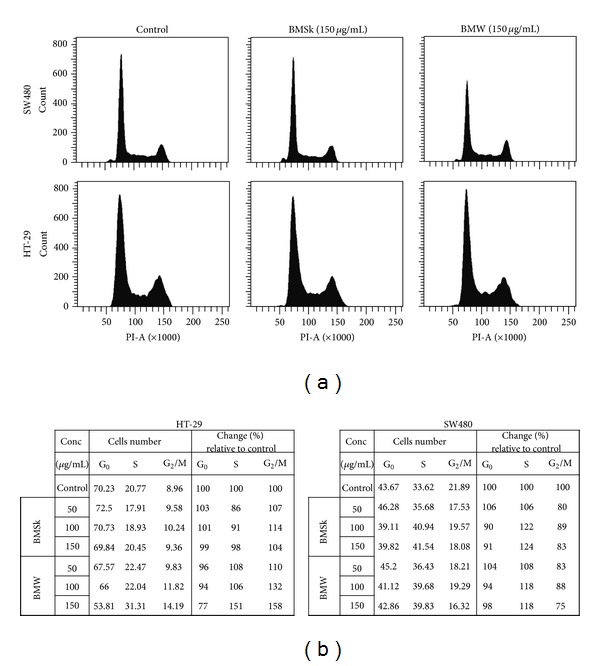
Bitter melon extracts cause S and G2/M cell cycle arrest. (a) Cell cycle analyses of bitter melon extract treated cells. HT-29 and SW480 cells were treated with 150 *μ*g/mL of bitter melon extract for 24 h, and subsequently examined by flow cytometry following propidium iodide staining for DNA content. Graphs are representative of data collected from three experiments. (b) Tabular representation of HT-29 and SW480 cells treated with 0 to 150 *μ*g/mL of bitter melon extract for 24 h.

To determine whether bitter melon extracts inhibit the cell cycle progression of HT-29 and SW480 cells, they were grown to 70% confluence and the cell cycle distribution was analyzed by flow cytometry after a 24 h exposure to 150 *μ*g/mL BMW and BMSk extracts. With HT-29 cells, both BMW and BMSk induced G2/M phase arrest (8.96% in controls versus 9.36 and 14.19% for BMSk and BMW, resp.) (Figure 2(a)). In addition, in HT-29 cells, BMW significantly induced S arrest, when compared to BMSk or control treatment (31.31% with BMW versus 20.45% and 20.77% for BMSk and control, resp.) (Figure 2(a)). In contrast to HT-29 cells, however, while both BMW and BMSk induced S phase arrest of SW480 cells, neither extract had an effect on the G2/M phase. To determine whether there is a critical amount of BMW extracts required for inducing the S- and G2/M arrest, we performed q dose escalation study. As shown in Figure 2(b), there was a dose-dependent increase in cell cycle arrest with both cell lines. Together, these studies demonstrate that BMW extracts are potent inhibitors of cell cycle, albeit in different manner depending on cell line.

### 3.3. Bitter Melon Extracts Do Not Induce Apoptosis

**Figure 3:**
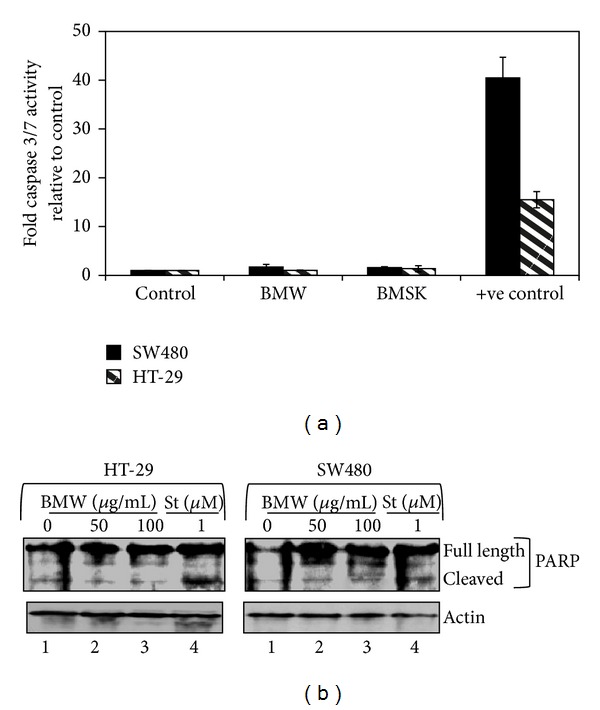
Bitter melon extract does not induce apoptosis in HT-29 and SW480 cells. (a) Bitter melon extract does not affect the caspase 3/7 activation, an apoptosis mediator. SW480 and HT-29 cells incubated with 150 *μ*g/mL of bitter melon extract were analyzed for apoptosis by caspase 3/7 fluorometric assay. Bitter melon extract treatment did not induce any caspase activity when compared to untreated controls. Staurosporine 1 *μ*M was used as a positive control. (b) Bitter melon extract does not affect the PARP cleavage in colon cancer cell lines. Western blot analysis for PARP cleavage of bitter melon treated cell lysates was performed using rabbit anti-PARP antibody. Bitter melon extract treatment resulted in no cleavage of PARP but significant PARP cleavage was observed in presence of a positive control, 1 *μ*M staurosporine (St). These data suggest that bitter melon extracts do not induce cells to undergo apoptosis.

We next aimed to determine the mechanism by which bitter melon extracts induced cell death. Given that previous studies have suggested that aqueous extracts of bitter melon induce apoptosis [15, 16], we performed caspase 3/7 assay as well as western blot analysis for apoptosis-related proteins. First, we determined the effect of the extracts on activation of effector caspases. Neither treatment with either BMW nor BMSk for 48 h induced caspase 3/7 activity (Figure 3(a)). Furthermore, there was no activation of Poly (ADP-ribose) polymerase (PARP), an established and reliable apoptosis indicator downstream of caspase activation (Figure 3(b)). To confirm the validity of the assay and the western blots, cells were also treated with staurosporine (1 *μ*M). In the cells, we found significantly increased caspase 3/7 activity and PARP cleavage. These data suggest that extracts from bitter melon does not induce apoptosis of these two colon cancer cell lines. 

### 3.4. Bitter Melon Extracts Induce Autophagy in SW480 and HT-29 Cells

**Figure 4:**
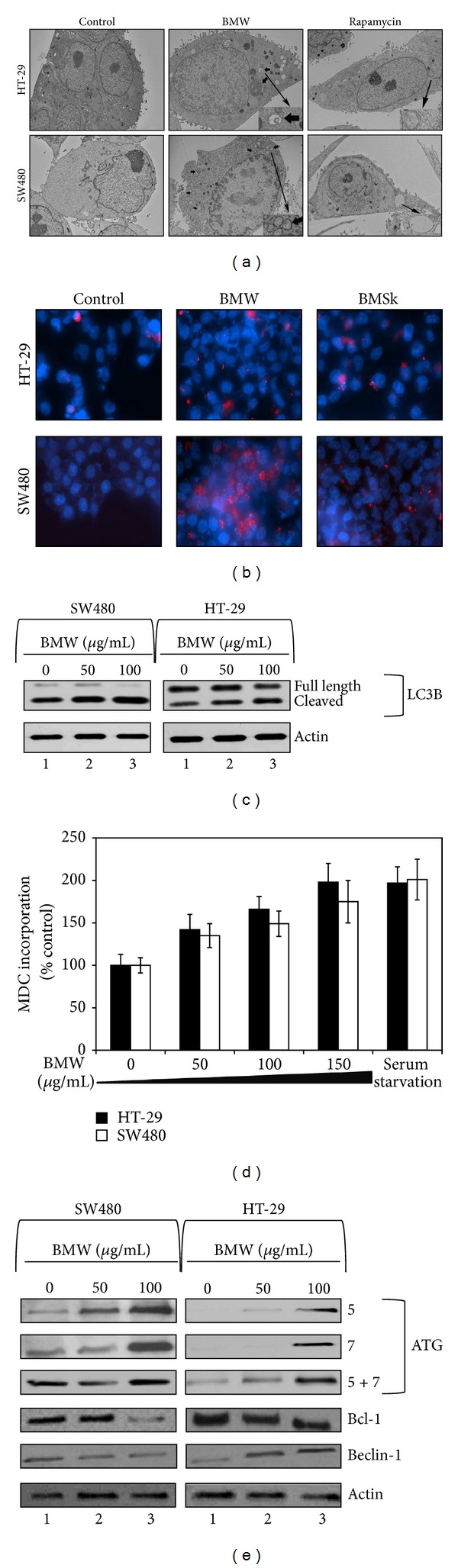
Bitter melon extracts induce autophagy in SW480 and HT-29 cells. (a) Electron micrograph of bitter melon treated SW480 and HT-29 cells. Investigation of ultrastructural morphology and autophagic vacuoles 12 h after treatment with 100 *μ*g/mL of bitter melon whole fruit extract. Rapamycin 25 *μ*M treatment for 12 hours was used as positive control. Insets in the TEM images show magnified autophagic vacuoles. Both SW480 and HT-29 show presence of autophagic vacuoles following treatments with BMW and rapamycin. (b) Effect of bitter melon extracts on LC3B cleavage. Immunocytochemistry analysis of SW480 and HT-29 cells treated with 100 *μ*g/mL of bitter melon whole fruit extract for 24 h shows enhanced accumulation of LC3B in cytoplasm. Immunostaining was performed using rabbit anti-LC3B antibody followed by Cyanine Dye (Cy3) labeled anti-rabbit IgG. This method only picks up cleaved LC3B. (c) Western blot analysis of SW480 and HT-29 cells treated with increasing concentration of bitter melon whole fruit extract showed increased levels of cleaved LC3B. (d) Monodansylcadaverine accumulation measured by fluorometric analysis showed increased accumulation of the dye within the cells treated with increasing concentration of bitter melon whole fruit extract for 48 h. For a positive control, the cells were serum starved for the same amount of time. (e) Western blot analysis for autophagy markers Bcl-2, Beclin-1, Atg 7 and 12. Both the cells lines treated with increasing concentration of bitter melon whole fruit extract showed decreased expression of Bcl-2 and increased expression of all the other markers in a concentration dependent manner.

Given that there was no apoptosis, we next determined whether the extracts could induce autophagy as a mechanism of cell death. A hallmark of autophagy is the generation of autophagosomes, which can be visualized by transmission electron microscopy (EM). Cells were treated for 24 h with BMW and then processed for EM. As shown in Figure 4(a), numerous autophagic vacuoles and empty vacuoles were observed in both SW480 and HT-29 cells treated with 150 *μ*g/mL BMW. Most of the autophagic vacuoles contained intact lamellar structure, cytoplasmic structure, and/or residual digested materials. On the other hand, tumor cells (SW480 and HT-29) treated with control media alone for 24 h did not exhibit autophagic features. As a positive control rapamycin (an mTOR inhibitor) was used which is well known for inducing autophagic vacuoles in colon cancer cells [22, 23]. 

To confirm that there was induction of autophagy, we determined the effect of the BMW extracts on activation of LC-3B, an ubiquitin-like protein. Pro-LC-3B is cleaved during the process of autophagy by the Atg4B protein in the cytoplasm to expose a C-terminal glycine residue resulting in the generation of the cytosolic LC-3BI form. The exposed C-terminus is then conjugated to phosphatidylethanolamine (PE) though an amide bond by a sequence of ubiquitination-like reactions [24]. The PE-conjugated form (LC-3BII) is tightly associated with the autophagosomal membrane [25]. To confirm that LC-3B is activated, we performed immunofluorescence studies. The two colon cancer cells were treated with BMW and then stained for cleaved (activated) LC-3B. Both cells treated with BMW extracts showed increased cytoplasmic accumulation of cleaved LC-3B as compared to untreated cells (Figure 4(b)). To further confirm LC-3B involvement we performed western blot analyses to observe whether LC-3B is cleaved in response to BMW treatment. In both HT-29 and SW480 cells, there was a significant dose-dependent increase in cleaved LC-3B (the 14 kDa LC-3BII isoform) following treatment with BMW extracts (Figure 4(c)). These results further suggest that BMW potentially induces autophagy in colon cancer cells.

To further confirm the formation of autophagic vacuoles upon BME treatment, we measured the incorporation of monodansylcadaverine (MDC), a marker for the acidic compartment within autolysosomes [26]. As shown in the bar diagram in Figure 4(d), there were significantly higher levels MDC incorporation in both SW480 and HT-29 cells following treatment with BMW extracts, the effect occurring in a dose-dependent manner. These results indicate that BMW extracts promote autophagy of colon cancer cells.

Beclin-1, also known as autophagy-related gene Atg6, is required for the initiation of the formation of the autophagosomes in autophagy [27]. Upregulation of Beclin1 has been shown to play a significant role in autophagic cell death. However, Beclin1 can be inhibited by interaction with anti-apoptotic protein Bcl-2 [27]. Hence, downregulation of Bcl2 has been shown to enhance autophagy functions. Accordingly, we determined the effect of BMW treatment on Beclin1 and Bcl2 expression. Western blot analyses of SW480 and HT-29 cells treated with BMW demonstrated increased expression of Beclin-1, while Bcl2 levels were downregulated (Figure 4(e)). To gain a better insight into the BMW induced autophagic pathways, we measured the effects of BMW treatment on the expression and translation of additional autophagy related marker proteins. Western blot analysis showed a marked increase in the expression of Atg 7 and Atg 12 genes in cells treated with BMW extracts (Figure 4(e)). Taken together, these data suggest autophagy as a potential mechanism for BMW extracts-mediated cell death.

### 3.5. Bitter Melon Extracts Affect Energy Homeostasis of Cancer Cells by Effecting Cellular ATP Though AMPK Mediated Pathway

**Figure 5:**
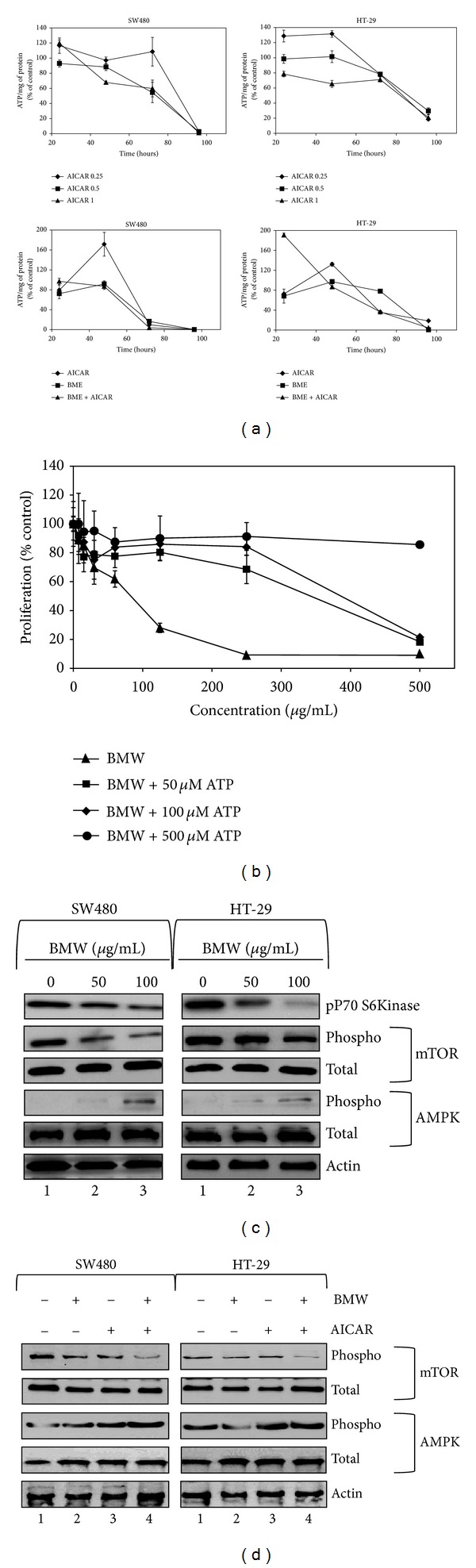
Bitter melon extracts affect energy homeostasis of cancer cells by effecting cellular ATP though AMPK-mediated pathway. (a) ATP modulation by bitter melon extract in HT-29 and SW480 cells. Cells were treated with increasing concentration of AICAR (AMPK activator), BMW (100 *μ*g/mL), or a combination of the two. AICAR treatment resulted in significant decrease in the ATP levels of the cells. A similar decrease was observed in the ATP levels upon BMW treatment. A more rapid decline was observed with the combination, at least in the initial time points. (b) Cell proliferation of BME treated cells was revived using exogenously added ATP. SW480 cells were treated with increasing concentration of Bitter melon extract (0–500 *μ*g/mL) with the media containing various concentrations of ATP (0–500 *μ*M). Exogenous ATP reversed the antiproliferative activity of BMW in a concentration dependent manner. (c) Western blot analysis for AMPK and its downstream regulators mTOR, and P70 S6Kinase. The two cells lines treated with increasing concentration of bitter melon whole fruit extract showed increased activation of AMPK, mTOR and P70 S6Kinase in a concentration dependent manner. (d) No additive effect in AMPK activation was observed by cotreatment with BMW and AICAR as compared to any one alone as measured via western blot. A significant additive effect observed in the activation of mTOR pathway by the combination suggesting BMW may activate mTOR by other pathways in addition to AMPK pathway.

To check whether BMW marked the colon cancer cells for autophagy by affecting energy homeostasis within the cells, ATP levels were determined for up to 96 h after treatment with 100 *μ*g/mL of BMW extracts. A time dependent decline in ATP level was observed in the cells (Figure 5(a)). Though there was greater decline in ATP levels at higher concentrations at 72 h versus 96 h of incubation, there was no marked difference in ATP levels from 12 to 24 h (Figure 5(a)). AMP activated protein kinase (AMPK) is a key energy sensor that maintains energy homeostasis [28]. AMPK promotes autophagy by directly activating Ulk1 [29]. To identify whether the alteration in ATP levels was due to an AMPK mediated mechanism, increasing concentrations of AICAR (0.25 to 1.0 *μ*M), a known inducer and activator of AMPK was added to the cells. A concentration and time dependent decrease in ATP levels, similar to that seen with BMW extracts, was observed with AICAR (Figure 5(a)) indicating an AMPK mediated mechanism. When BMW extracts and AICAR were used at maximum concentrations, no additive effects were observed further suggesting that BMW works though AMPK mediated pathway (Figure 5(a)). We next checked whether the decrease in ATP levels results in reduced cell proliferation. Studies were performed with exogenously added ATP. BMW-mediated suppression of proliferation of both SW480 and HT-29 cells was reversed with the exogenously added ATP in a dose-dependent manner (Figure 5(b)). When exogenous ATP concentrations reached 500 *μ*M, proliferation of BMW extracts treated cells was similar to that of control, untreated cells (Figure 5(b)).

Given the changes in ATP levels and the effect of AICAR, western blot analysis was additionally carried out to confirm whether the BMW extracts activated AMPK. Treatment with BMW extracts resulted in a significant increase in phosphorylated AMPK levels in a dose-dependent manner. There were no changes observed with total AMPK. Additionally, a marked decrease in the intensity of the phosphorylation of mTOR and p70S6K was also observed showing that the extract is causing the autophagy though an AMPK facilitated, mTOR mediated pathway (Figure 5(c)). To further confirm whether the AMPK and mTOR activity were not independent effects of the extract treatment, the cells were further treated with AICAR and BMW individually as well as in combination and its effect on the proteins was determined. Western blot analyses demonstrated that AMPK activation in the SW480 and HT-29 cells does lead to mTOR mediated signaling as mTOR was activated both in the presence of AICAR as well as BMW. Further there was an additive effect observed when combination of BMW and AICAR was used at their IC_50_ values (Figure 5(c)).

### 3.6. Bitter Melon Extracts Possess Anticancer Stem Cell Activity

**Figure 6:**
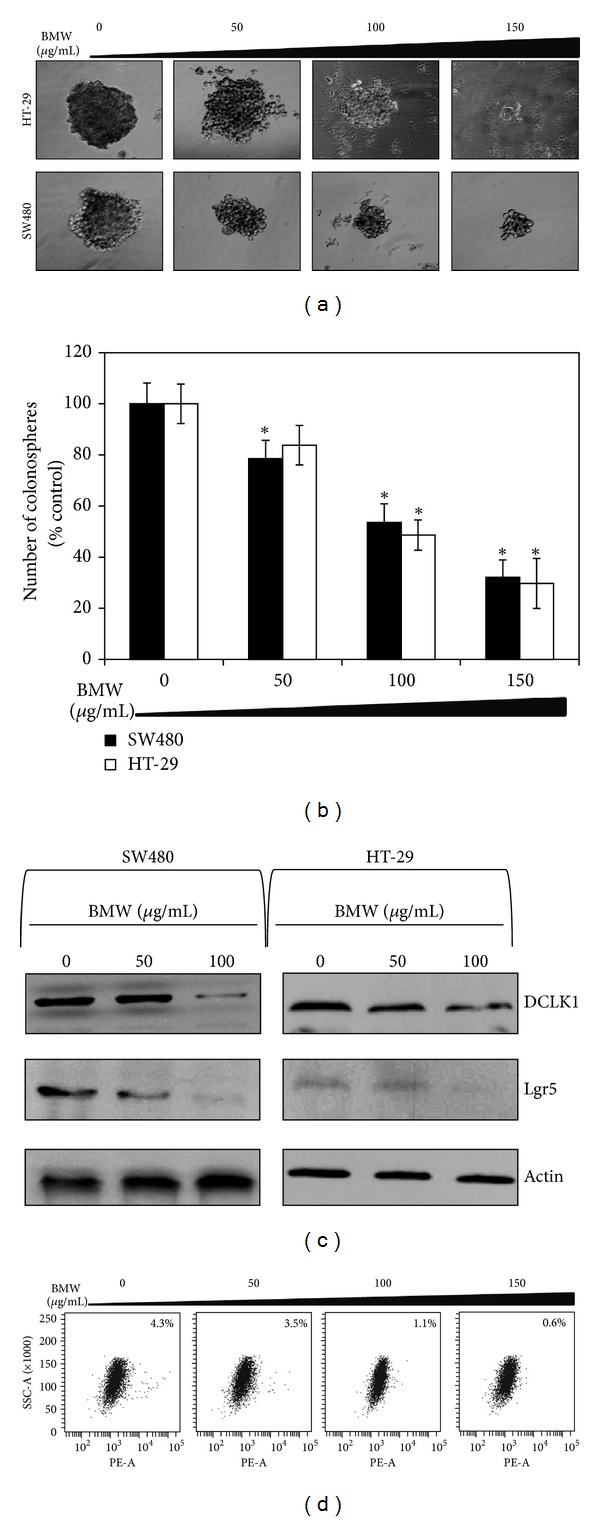
Bitter melon extracts possess anticancer stem cell activity. Bitter melon extract affects cancer stem cells. (a) SW480 and HT-29 cells were grown in specific spheroid growth media in low adherent plates and treated with increasing dose of bitter melon extract. After one week, the colonospheres were photographed and counted. (b) Graphical representation of the number of colonospheres. Bitter melon extract significantly inhibited colonosphere formation (*P < 0.05). (c) Western blot analyses of both cell lysates treated with bitter melon extract showed significant reduction in cancer stem cell markers proteins DCLK1 & LGR5. (d) Sorting of SW480 cells using anti-DCLK1 antibody by flow cytometry. 24 h after treatment, bitter melon extract caused significant reduction in the number of DCLK1 expressing cells in a concentration dependent manner.

Recent studies have demonstrated that a small population of cells contains tumor initiating potential while the majority of cells within a tumor have undergone differentiation and lost this potential [30]. Colonospheres are spheroids that are grown in ultralow binding plates and are believed to represent the growth of cells from stem cells [31]. Hence, the colonosphere cultures are used extensively to determine the effect of agents on stem cells. Accordingly, to determine the effect of bitter melon extracts on 3D cultures, cells treated with BMW were used for spheroid formation. The BMW-treated cells showed marked decrease in spheroid formation, when compared to control-treated cells (Figure 6(a)). Moreover, BMW treatment resulted in a concentration dependent decline in both the size and number of spheroids formed (Figure 6(b)). 

We have recently demonstrated that Doublecortin Calmodulin-like Kinase 1 (DCKL1) is a marker of quiescent stem cells in a variety of cancers including colon cancers [32]. Since, BMW extracts inhibited colonosphere formation, we determined whether DCLK1 expression was affected. Western blot analyses demonstrated that DCLK1 expression was markedly reduced in cells treated with BMW extracts (Figure 6(c)). Another stem cell marker in the colon Lgr5 was also studied to further confirm the activity of BMW on colon cancer stem cells. Similar to DCLK1, there was a concentration dependent decline in Lgr5 expression in BMW-treated cells (Figure 6(c)). Further confirmation was obtained by flow cytometry, where a significant reduction on DCLK1+ cells was observed in cells treated with BMW extracts (Figure 6(d)). These data suggest that BMW extracts are potent inhibitors of colon cancer stem cells.

## 4. Discussion

The data presented in this paper demonstrates that the methanolic extract of bitter melon is potent in inhibiting the growth of colon cancer cells. Since, cancer is a disease involving uncontrolled proliferation of cells, if a drug product is able to deter this cell division, it can potentially possess anticancer activity. Given the potent inhibition of proliferation of HT-29 and SW480 colon cancer cells by the bitter melon extracts, we proceeded with determining the mechanism of action. As a first step, we tested whether the activity resides in the skin or in the flesh. We did this because previous studies have demonstrated potent anticancer activity in the skin of grapes and peanuts [33, 34]. In bitter melon, our data suggests that the active ingredient most probably resides in the flesh as it inhibited cell proliferation with a much higher efficiency than the extracts from the skin.

A critical piece of information gathered from these studies is that there was a concentration dependent increase in cell cycle arrest in the S phase for both cell lines. More importantly, the cell cycle arrest was found to be more significant in extracts from the flesh as compared to the skin. Previous studies suggest that S phase arrest in cell cycle may represent eventual cell death by autophagy [35]. In this regard, the extracts did not induce apoptosis. Although there was a reduction in the antiapoptotic protein Bcl2, there was no activation of PARP or caspases. However, there was an increase in the autophagy-inducing protein Beclin-1. This was further conformed by electron microscopy, which showed the presence of autophagic vacuoles with their typical double membrane structure. Presence of autophagosomes is a hallmark of autophagy. 

LC3B cleavage is another strong indicator of cells undergoing autophagy. During autophagy LC3B is released which then binds with autophagic vacuoles [25]. Immunocytochemistry analysis showed significant increase in the expression levels of LC3B. Further, the increased levels of cleaved LC3B as observed by western blot indicate LC3B activation showing presence of active autophagy. Another indicator of autophagy is though the increased incorporation of MDC in the cells. MDC is a specific *in vivo* marker for labeling autophagic vacuoles [26]. Again, there was a marked increase in the incorporation of MDC in the cells treated with bitter melon flesh extracts. Furthermore, the levels of Atg 5 and Atg 7, two well-known autophagy markers significantly increased in the treated cells further confirming the presence of autophagy as the mechanism of death for the cells. 

Although BMW affected SW480 cells only at the S phase, in HT-29 cells we observed both S- and G2/M arrest. Although the reasons for this difference are currently unknown, the one thing to think about would be whether there are differences related to genotypes. In this regard, a recent manuscript suggests that HCT-116 cells, also a colon cell line undergoes apoptosis when exposed to bitter melon aqueous extracts, while our studies suggest that the cells undergo autophagy [36]. One thing that is obviously different between HCT-116 and the two cell lines used in this manuscript is the p53 status. While HCT-116 cells are wild type for p53, both SW480 and HT-29 cells have a mutation. Further studies are necessary to determine whether p53 status has any effect in response to treatment with bitter melon extracts. 

Though recent studies have reported a potential role of bitter melon in both preventing as well as attenuating the cancer progression, its potential mechanism of action has not been properly elucidated [15, 16]. Bitter melon has been used conventionally as an antidiabetic phyto-product in Ayurvedic and Traditional Chinese systems of medicine. The mechanism by which most antidiabetic medications work is either by acting as an insulin mimetic or by affecting the energy homeostasis of the cells by modulating the metabolic pathways. Bitter melon has been suspected to utilize the second strategy and our results suggest the same. Some energy modulators as well as calorie control in cancer patients have been shown to have positive effect on tumor progression [37]. Upon treatment of colon cancer cells with increasing concentration of bitter melon flesh extracts, there was a marked decrease in ATP production. This reduction in ATP production was similar to the ATP reduction seen with AICAR (a known AMPK activator). Further confirmation that the energy modulating activity of bitter melon extracts is in part responsible for cell death was obtained when ectopically added ATP was able to partially rescue the antiproliferative activity. 

AMPK is a well-known energy sensor within the cells and has been previously implicated in inducing either apoptosis or autophagy upon its activation [38, 39]. Indeed, cells treated with the bitter melon extracts resulted in the activation of AMPK. Previous studies have demonstrated that AMPK has been known to modulate autophagy via an mTOR-mediated pathway, which was also observed in our current study. As expected, the bitter melon extracts resulted in marked increase in the activation of mTOR. This activation of mTOR was further enhanced by the addition of AICAR. In the ATP estimation assay where maximally active doses of both bitter melon extracts and AICAR were used, the cotreatment did not result in any added effect. Similarly during the western blots when the IC_50_ concentrations of both the therapeutics were used there was an additive effect on the protein activation. 

It is being increasingly understood that there are a rare population of cells within a tumor that have the capacity to initiate and sustain tumorigenesis. These cancer stem cells, also termed cancer-initiating cells exhibit properties such as drug resistance and label retention, and are phenotypically undifferentiated. Stem cells have been increasingly recognized as the cause for not only primary tumorigenesis, but also for relapse of a tumor. A method of growing cells that represents growth from the stem cells is the colonosphere assay, where the cells are allowed to grow and form spheroids in ultralow attachment plates. So for a therapeutic entity to be a successful anticancer agent it needs to be effective against cancer stem cells, and reduction in spheroid formation capacity is used as one measure. Similar to that seen with proliferation assays, the effect of bitter melon flesh extracts was again found to be much stronger than the skin extracts in colonosphere assays. The size and number of the spheres were found to significantly decrease in cells treated with the extracts from the flesh as compared to control cells, indicating that the extracts can potentially act against cancer stem cells limiting their propagation and proliferation. 

Markers for the identification of the stem cells are being debated, at least in the normal intestinal mucosa [40, 41]. There have been multiple proteins that have been identified as stem cells markers, but the ones that have been in the forefront are LGR5 and DCLK1, although other proteins such as Bmi1, hTert, and OLFM4 have also been proposed [42–44]. Studies on colon cancer, however, have been much more limited but equally controversial. For example, a cell surface marker CD133 has been extensively used for studies related to stem cells but subsequent studies has demonstrated that both CD133+ and CD133− population of cells have equal propensity to form tumors [45]. Our laboratory has focused on DCLK1, a calmodulin-like kinase not only because it was originally identified to mark quiescent stem cells in the normal intestine, but also that it is a marker for colon cancer stem cells [46]. In the current study, we have observed that bitter melon extracts reduce the number of DCLK1+ cells in a dose-dependent manner, suggesting that the extracts target the stem cells. Another potential cancer stem cell marker Lgr5 [47, 48] was also studied and found to be affected in similar manner to DCLK1. Further studies are necessary to determine whether the mechanism of action for the extract in inhibiting the stem cells is the same as that seen with the fast dividing progenitor cells. These data suggest that bitter melon extracts can potentially be an effective therapeutic against colon cancer by acting against both the rapid proliferating cells as well as the cancer stem cells in colorectal cancer. 

In conclusion, the results of the present study indicate that extracts from the flesh of bitter melon can actively inhibit the growth of colon cancer cells *in vitro *by inducing autophagy. Therefore, bitter melon extract can be utilized as a chemopreventive/therapeutic agent in colon cancer. 

One puzzling piece of information that remains to be teased out is that the current studies showed efficacy of methanolic extracts and not aqueous extracts of the fruit. This is in contrast to previous published studies demonstrating efficacy of aqueous extracts against prostate and breast cancers. The reasons for the differences in activity are currently unknown, but there are various possibilities. Of course, as mentioned above, one major difference in the two studies is the type of extracts used. The method of preparation can lead to differences in the overall content of the extracts and hence in differences in mechanism of action. We have utilized a methanolic extract, while previous studies used aqueous extract [14]. Surprisingly, in our studies, aqueous extracts were not as potent as methanolic extracts on colon cancer cell lines. A second reason could be the source of the fruit. We have used *Momordica charantia* Linn, Indian subcontinent variety. It is not clear what variety was used in the previous studies. Our future studies will involve testing various varieties of bitter melon, including the Chinese and Vietnamese varieties. A third reason could be organ specificity of the extracts. It is possible, that various extracts may act in different ways on cells from different organs. This will need extensive, careful, additional studies, which we hope the reviewers agree is outside the scope of the current manuscript. Finally, the effects can be due to genotype variations in the cells. It is possible that there can be differential response of cancer cells to compounds or complex mixtures based on their genetic make up. Mutations, overexpression, or deletions of various genes can alter response by altering DNA repair pathways or cell signaling. This could also be the reason for autophagic effects of the bitter melon extracts in colon cancer cells. More studies are therefore needed to check whether the p53 (guardian of the genome) status of the cells or the DNA repair defect status of the cells (common mutations in colon cancers) can lead to differential response to bitter melon extracts. Additional purification studies are also warranted to identify the active compound(s) in the extracts. Once such compounds are identified, a cocktail of these compounds could be used to effectively target cancers in the preventive and therapeutic setting.
